# Retrospective four‐dimensional magnetic resonance imaging of liver: Method development

**DOI:** 10.1002/acm2.13108

**Published:** 2020-12-03

**Authors:** Henna Kavaluus, Tiina Seppälä, Lauri Koivula, Eero Salli, Juhani Collan, Kauko Saarilahti, Mikko Tenhunen

**Affiliations:** ^1^ Comprehensive Cancer Center University of Helsinki and Helsinki University Hospital Radiotherapy Finland; ^2^ Department of Physics MATRENA doctoral programme University of Helsinki Helsinki Finland; ^3^ Medical imaging center, Radiology University of Helsinki and Helsinki University Hospital Finland

**Keywords:** liver, radiotherapy, retrospective 4D‐MRI, SBRT

## Abstract

Purpose of our research was to develop a four‐dimensional (4D) magnetic resonance imaging (MRI) method of liver. Requirements of the method were to create a clinical procedure with acceptable imaging time and sufficient temporal and spatial accuracy. The method should produce useful planning image sets for stereotactic body radiation therapy delivery both during breath‐hold and in free breathing. The purpose of the method was to improve the localization of liver metastasis. The method was validated with phantom tests. Imaging parameters were optimized to create a 4D dataset compressed to one respiratory cycle of the whole liver with clinically reasonable level of image contrast and artifacts. Five healthy volunteers were imaged with T2‐weighted SSFSE research sequence. The respiratory surrogate signal was observed by the linear navigator interleaved with the anatomical liver images. The navigator was set on head‐feet — direction on the superior surface of the liver to detect the edge of diaphragm. The navigator signal and 2D liver image data were retrospectively processed with a self‐developed MATLAB algorithm. A deformable phantom for 4D imaging tests was constructed by combining deformable tissue‐equivalent material and a commercial programmable motor unit of the 4D phantom with a clinically relevant range of deformation patterns. 4D Computed Tomography images were used as reference to validate the MRI protocol. The best compromise of reasonable accuracy and imaging time was found with 2D T2‐weighted SSFSE imaging sequence using parameters: TR = 500–550 ms, images/slices = 20, slice thickness = 3 mm. Then, image processing with number of respiratory phases = 8 constructed accurate 4D images of liver. We have developed the 4D‐MRI method visualizing liver motions three‐dimensionally in one representative respiratory cycle. From phantom tests it was found that the spatial agreement to 4D‐CT is within 2 mm that is considered sufficient for clinical applications.

## 1 Introduction

### 1.1 Stereotactic radiotherapy of liver and medical imaging

Stereotactic body radiotherapy (SBRT) is targeted to small tumor volumes in body area and is typically applied in lung lesions and increasingly in prostate. Small liver lesions are also suitable targets of SBRT.^1^ New evidence of the benefits of higher radiotherapy (RT) doses to small liver metastases compared with traditional lower dose levels of palliative RT indicates a future increase in the need of liver SBRT. With SBRT, it is required to contour target accurately and tumor physiological motion needs consideration in SBRT treatment.

Motion of the liver is mainly caused by three separate factors: respiration, random peristaltic motion, and pulsatile cardiac motion.^2^ While respiratory motion can affect a relatively large portion of the liver, cardiac‐induced motion of the liver is mainly found in the area underneath the heart.^3^ Respiratory‐induced motion is continuous and repeated, which enables averaging the motion of the whole liver. Liver tissue is deformable; as these separate motion forces affect from different directions, the resultant motion pattern in different parts of liver will also be complex.

The SBRT treatments can be delivered either with reduced respiratory motion or with free breathing. The reduced respiratory treatment can be delivered either with breath‐hold or with abdominal compression.^4^ Sometimes, treatment with breath‐hold or with abdominal compression is impossible for multiple reasons. A patient may be incapable of repeating the breath‐hold instructions, which would lead to treatment being delivered in free breathing during all or parts of the patient’s breathing phases. Subsequently accurate estimation of target motion is required to define comprehensive margin coverage to the clinical target volume.

Computed tomography (CT) images, with or without contrast agent, are current standard with supplementary co‐registered MR and/or PET images in radiotherapy planning (RTP) to contour the target volumes. Dose planning and calculations are mostly made based on the CT images. During RT, the patient is set up to the correct treatment position by using the CT images as a reference image to cone beam CT images (CBCT) of image guidance. However, a single CT image series does not model the motion of tissues and has inferior soft‐tissue contrast than magnetic resonance images. In CBCT, the liver lesions are poorly visible.

Magnetic resonance imaging (MRI) has become the general modality for delineation purposes of liver tumors.^5^, ^6^. Image quality affects the quality of delineation and thus needs consideration.^7^ MR images have much better soft‐tissue contrast, but it has more challenges with the imaging of moving objects. The moving objects are usually imaged using breath‐hold and/or triggered imaging (expiration). The MRI is time‐consuming and therefore image quality suffers from motion artifacts especially when imaging the abdomen area. The purpose of this work was to overcome these limitations of MRI in moving objects and produce similar 4D dataset as from CT but with improved soft‐tissue contrast.

### 1.2 Imaging method of predicting motion of liver

Four‐dimensional CT (4D‐CT) has been routinely used to track the motion of gross tumor volume (GTV) in lung areas. Tumors in lung areas can be observed from CT images even with CT’s poor soft‐tissue contrast. Even with the contrast agent, the visibility of the tumor in the upper abdomen often remains insufficient. The 4D images probabilistically visualize the average motion of the tissues. The 4D images are based on the 3D image volumes that are displayed as a function of breathing phases. The 4D‐CT images are reconstructed by tracking the respiratory signal and reconstructing the dynamic images with different methods. The motion information of the GTV is used to define comprehensive margin coverage to internal target volume (ITV).^8^

A 4D‐MRI method is required to integrate the characteristics of 4D imaging and great soft‐tissue contrast of MRI. 4D‐MRI is utilized to build 4D model of liver. The model may be used to define comprehensive ITV margins and to choose best breathing phase or phases for SBRT treatment delivered. In addition, the model can be utilized to build deformable liver model.

There are several publications about predicting the motion of an abdominal area reviewed comprehensively by Stemkens et al. (2018).^2^ There have been developed prospective and retrospective methods of 4D‐MRI of liver. There are publications with 2D and 3D MRI with cine,^9^ interleaved,^10^ and sequential acquisitions.^11^ The main respiratory‐induced displacement in liver is in cranio‐caudal (CC) direction and it can be up to several centimeters.^2^, ^10^, ^12^ The liver additionally shows rigid 1–12 mm anterior–posterior (AP) and 1–3 mm left–right (LR) transformations. In addition to the rigid transformations, there occur also nonrigid deformations in liver tissues (up to 20 mm).^13^ Uh et al. (2017)^14^ has also researched relations between organ motion and specific patient characteristics.

2D MRI acquisition can be made in either coronal, transversal, or sagittal plane and the other two planes are reconstructed. There are publications of 4D‐MRI methods in all three 2D acquisition planes.^2^ Transversal acquisition plane is the most natural orientation for delineation purposes, since it is most commonly used in RTP.^2^ Van De Lindt et al. (2016)^9^ chose sagittal plane acquisition to their research, since in the coronal motion quantification, the out of plane AP motion is a potential cause of registration error. Transversal plane was excluded, since motion in SI direction results in large amount of through plane motion for transversal plane and spins of moving tissue may therefore move from one slice to the next causing image artifacts. According to Liu et al. (2014),^15^ acquisition in sagittal plane is often optimal in order to minimize though plane motion since left–right motion is generally smallest. There are increased number of 3D methods published. Stemkens et al. (2018)^2^ has reviewed the published 3D methods.

External, internal surrogate respiratory signal^16^ and self‐navigation approaches have been utilized to reorder 4D‐MRI data. Nehrke et al. (2000)^17^ published that pencil beam navigator pulses can be used to represent *in vivo* motion. In addition, Stemkens et al. (2015)^18^ published that the navigator echo is more reliable for sorting 3D MRI volumes than the respiratory bellows. There are multiple different methods for sorting slices: sorting k‐space or images, amplitude binning, or phase binning. Amplitude binning sorts respiratory data in N number of bins based on the amplitude of signal and phase binning sorts each respiratory cycle in N number of bins. Generally, the number of bins is 4–10 but 4D‐CT generates 10 bins that it is used in most of the published studies. Phase and amplitude binning can be used as combination. Respiratory data are sorted at first in phases and predefined amplitude range is chosen to the final 4D image.^11^ There are also approaches of collecting data or modeling only part of the breathing phase such as mid‐ventilation^9^ and mid‐position^19^ methods. Usually sorting is made in one breathing cycle, which simplifies the breathing. In addition, sorting can be made over tens of minutes to time resolved 3D to study irregularities in organ motion during free breathing^10^ All retrospective methods, however, have the disadvantage that the sequence is agnostic of the respiratory waveform during the acquisition.^2^

The aim of this research was to develop a retrospective 4D‐MRI protocol for clinical use in the liver SBRT. The research is focused to optimize accurate MRI method and data processing methods. The developed method is tested and evaluated with self‐manufactured 4D phantom.

## 2 Methods

### 2.1 Prerequisites for a 4D‐MR imaging protocol of liver

**Fig 1:**
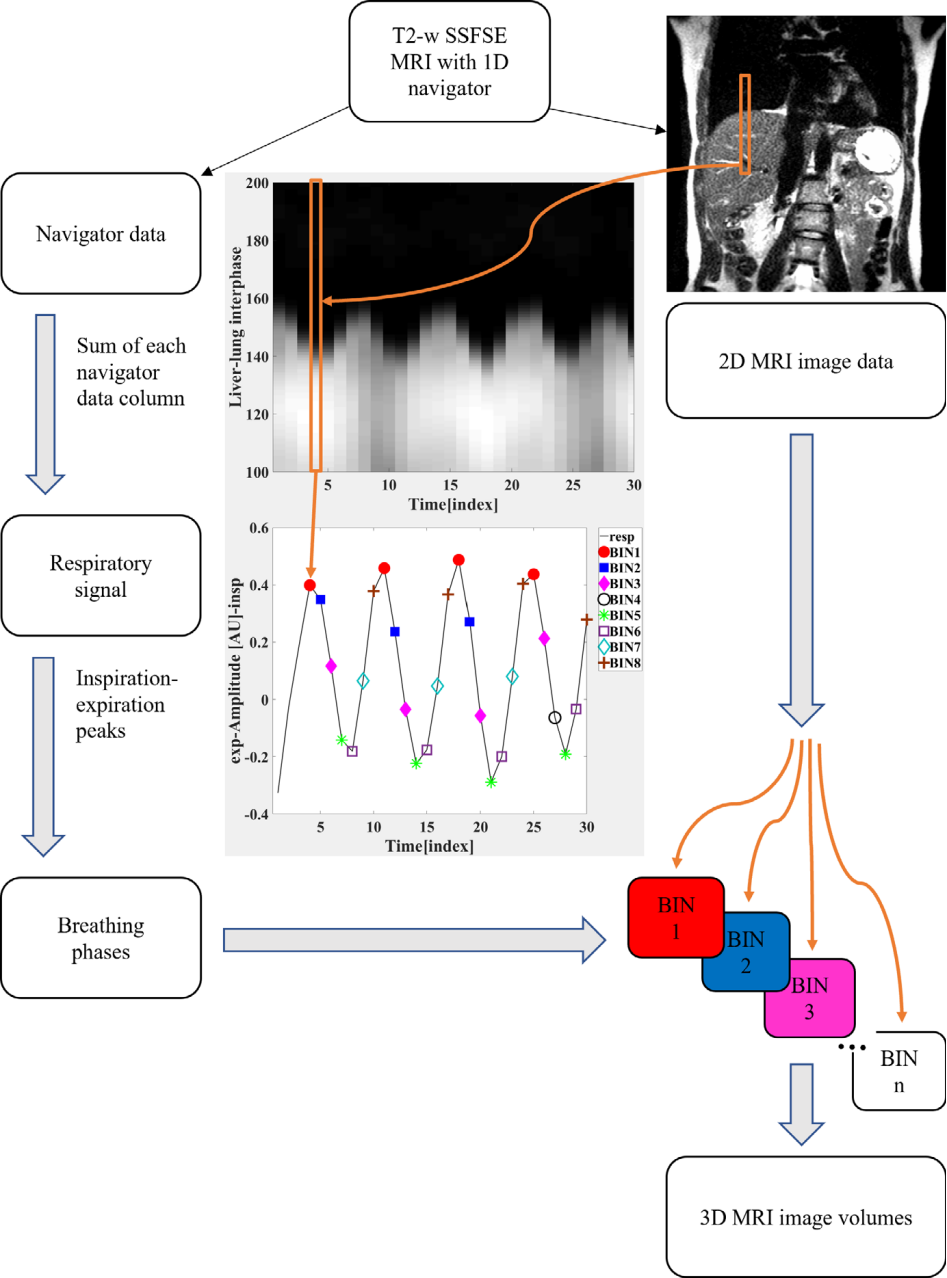
A diagram of the workflow of the developed method. Volunteers were imaged with T2‐w 2D SSFSE MRI sequence interleaved with linear navigator echoes. Navigator echoes were used to collect 1D image data to get the position of liver–lung interface. The 2D MR images and navigator data were retrospectively processed with self‐developed MATLAB algorithm. Navigator data were used as respiratory surrogate signal and the data were sorted into breathing phases. The 2D image slices of liver were sorted into N number of bins according to breathing phases. Respiratory‐induced liver motion was observed from resulted 4D‐MR images. The 4D images are used to track the motion of small liver lesions and to build deformable liver model for SBRT use

The 1.5 T MRI scanner (Optima MR450w GEM, GE Healthcare, Waukesha, WI, USA) equipped with the GE oncological package was utilized in the study (see workflow in Fig. 1). A T2‐weighted (T2‐w) single‐shot fast spin echo (SSFSE)^20^ research sequence was carried out with the interleaved navigator echoes localized at the diaphragm. The T2‐w MRI was performed for the upper abdomen area to study liver motion as a function of time. The T2‐w SSFSE has clinically accurate soft‐tissue contrast in liver area.^11^ Navigator echoes were used to collect 1D image data to get the position of liver–lung interface. The navigator was mounted and centered according to the 2D localizer images on the dome of the liver to observe the motion range of the diaphragm. Each 1D navigator image corresponds to one T2‐w SSFSE image slice.

At first, we tested the clinical cine MRI by the FIESTA (bSSFE) sequence to acquire liver motion. With the 1.5 T MRI scanner, the cine imaging has unreasonable long imaging time for our purposes (imaging time would be at least 40 min with 3 mm slice thickness). Therefore, we used the investigational 4D SSFSE sequence with acquiring time of 10–15 min. The image quality was partly better in the SSFSE sequence compared to the FIESTA sequence; there occurred less artifacts, like banding.

Imaging parameters were optimized according to clinical purposes and requirements of the developed method. The optimal imaging method should be clinically usable; reasonable long imaging time and the quality of the images at the reasonable level. MR images are being used in order to determine liver motion three‐dimensionally during the whole respiratory cycle with sufficient accuracy. In order to get a high‐quality 4D liver model, each anatomical position needs to be covered over the full respiratory cycle.

MR images must have acceptable resolution (3 mm, mentioned in the reference [15]) and low number of artifacts in the acquisition direction. Artifacts were minimized with fixation in RT treatment position to minimize extra motion. RT treatment position typically requires a flat tabletop, MR compatible fixation and lasers.

### 2.2 Principles of model

MR images and navigator data were processed retrospectively by a self‐developed MATLAB (MATLAB (2016a), The MathWorks, Inc., Natick, Massachusetts, United States) algorithm. The image elements of navigator signal were classified to “high‐signal elements” (liver) and “low‐signal elements” (lung) by thresholding the signal. The threshold values were defined for each navigator dataset individually since the image intensities were varying in each dataset. A time‐dependent respiratory signal was reconstructed by counting the number of “high‐signal elements” in each column of the navigator data. The number of “high‐signal elements” in one column corresponds to the diaphragm position at that time point.

The sum of one column corresponds to the magnitude of the diaphragm position (in arbitrary units) at that time point. The respiratory signal was then used to retrospectively sort the MR image data into the selected number of breathing phases.

The inspiration and the expiration phases were detected from the respiratory signal that was divided into single respiratory cycles according to the time points of end‐inspiration. End‐inspiration locations were detected by finding where the derivative of the respiratory signal changes from positive to negative. The desired model is to follow average motion of the liver and therefore, the breathing instructions were excluded to avoid affected or other unrelaxed breathing. Respiratory cycles were sorted in respiratory phases (bins) by phase binning. The phase binning gives probabilistic information about the spatial state of the liver. Phase binning divides one respiratory cycle into number of bins (N) in time domain. The number of data points in one cycle defines the quality of phase binning.

The number of bins were optimized to reduce intra‐bin variability and amount of missing data. The intra‐bin variability occurs if one bin consists of data points from wide amplitude range of the respiratory signal. To avoid the intra‐bin variability effect, it is possible to set the number of the bins close to number of the data points in one breathing cycle. In addition, there are intra‐bin variability caused by multiple suitable slices per location at one bin. The effect is caused by data sampling. It is not suitable to choose outlier respiratory signal points to our model since very deep inspirations and expirations are more infrequent events than the normal inhale–exhale amplitude of the mean respiratory signal. The slice imaged with minimum difference between the amplitude and mean amplitude of the corresponding bin was chosen to the final 4D‐MR image. The similar strategy was also introduced in literature.^11^

If no suitable slice has been found for a certain bin, the missing data effect occurs from sampling the data and it leads to empty bin and thus empty slices to the final 4D‐MR image set. Missing data problem cannot be fully avoided, but it can be reduced by increasing the imaging time, decreasing the matrix size in phase‐encoding (PE) direction, or decreasing the number of bins.

**Fig 2:**
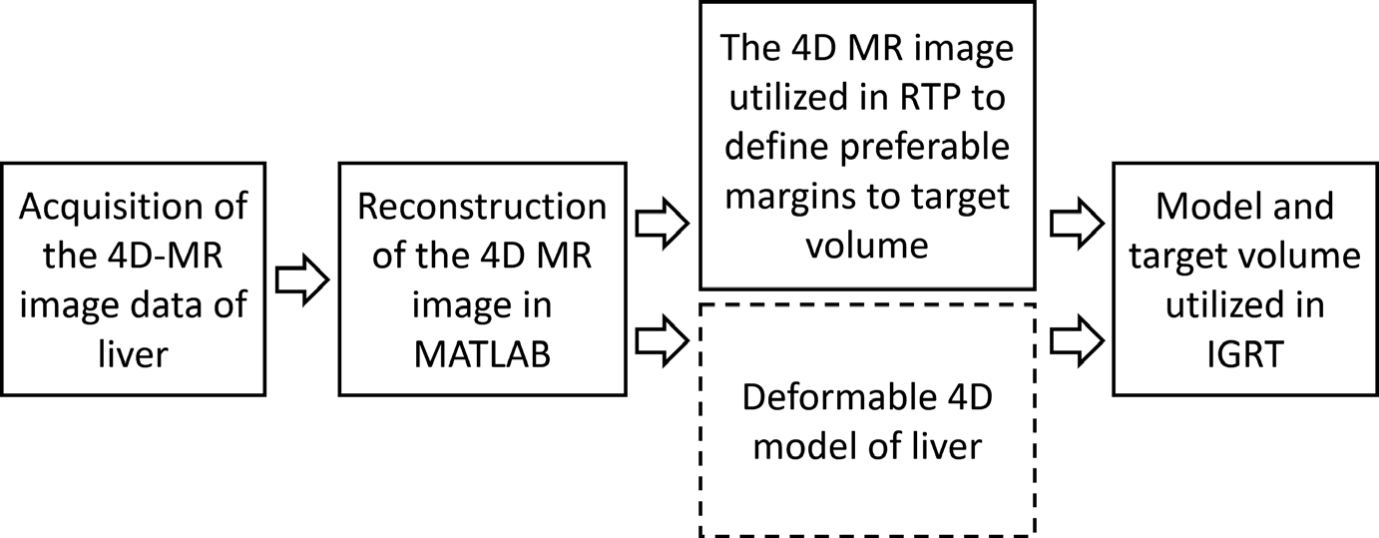
A diagram of the proposed workflow of the methodology. At first, the liver is imaged with the 4D‐MRI sequence. Secondly, the MR images are sorted with self‐developed algorithm in MATLAB to reconstruct the dynamic 4D‐MR image. The 4D‐MR image is utilized in radiotherapy planning to get more accurate CTV to ITV margins. In addition, the 4D‐MR image is utilized to create deformable liver model that will be introduced in our future studies. Prefer treatment volumes and deformable liver model will be utilized in image‐guided radiotherapy (IGRT)

Slices sorted into bins were used to reconstruct 3D volume images to each phase. The empty slices in 4D‐MR image were replaced with slice from previous bin at the same image location (nearest neighbor). The time it takes to process all the images was approximately 5–15 min and depends on the quality of the navigator data and power of the computer utilized in computation. A diagram of the proposed workflow is presented in Fig 2.

### 2.3 Optimization of parameters

The 4D‐MRI sequence^20^ was developed and optimized with imaging five healthy volunteers (two males and three females, with range of age 27–52 years) using an investigational imaging sequence (2D SSFSE interleaved with linear navigator) in the 1.5 T GE Optima 450w GEM scanner. Our institutional ethics committee approved the trial protocol (HUS/395/2018). The volunteers provided written informed consent before initiation of the study.

Motion artifacts are minimized with RT fixation. Image contrast was improved with surface coils. Volunteers were fixed with footrest in‐feet‐first‐supine orientation. A pillow was set under volunteer’s head and arms were raised above the pillow to a routine RT imaging position of abdomen. The anterior array (AA) surface coil was utilized with posterior array to use 36–48 channels in field of view (FOV). The AA coil was set on the surface of the abdomen and supported by pillows to prevent the weight of the AA coil on volunteer. At the first imaging experiment FOV was 40 cm, but it was later adjusted to consist of the whole liver area of the healthy volunteer and therefore it was different in every MR image set depending on the size of the subject (healthy volunteer).

Imaging directions were optimized to get two orthogonal views, which enables true 3D tracking of the liver. Five healthy volunteers were imaged in coronal and in transversal imaging planes and two volunteers were imaged additionally in sagittal plane. Conventional RTP utilizes CT images, which are imaged in transversal imaging plane. Transversal imaging plane was chosen to MRI protocol because the images can be compared with CT images. The coronal and sagittal imaging planes were compared together. Sagittal imaging plane was excluded since the total imaging time was longer than in coronal imaging plane.

Imaging time was optimized for clinical protocol since the time consumed for MRI should be as short as possible. Von Siebenthal et al. (2007)^21^ published that it is not appropriate to increase the imaging time too much when there is a small drift of liver over time caused by gravity, bowel movement, and muscle relaxation. The drift changes continuously the position of the liver. Long imaging time is also a cost for MRI resources and therefore the imaging time should be increased only with appropriate reason. Conventional imaging time in one clinical MRI sequence is from 2 to 15 min and it should not be substantially extended. The MRI has several parameters to optimize, which all effect to imaging time. Main parameters are TR, TE, number of images/slices, slice thickness, and number of voxels in PE direction.

TE value was set constant (80 ms) to get T2‐w MR images. TR value was optimized testing with multiple values (400–1500 ms). With low TR, there occurred some artifacts in navigator data, which disturbed the data analysis. With higher TR, imaging time was increased, and sampling became finally unreasonable low. Variable flip angles, α were used in the SSFSE sequence^20^ with parameters α_init_ = 130°, α_min_ = 90°, α_cent_ = 100°, and α_last_ = 45°.

Number of slices per location was optimized between the values 5 and 20. Multiple slice thickness and the quality of images were compared between 5, 3, and 2 mm slice thicknesses. The target volumes of liver SBRT are typically metastases with dense vasculature. In our healthy volunteers there were no real liver lesions so we decided to use visibility of small veins as surrogates for target objects.

Number of voxels in PE direction was set to the minimum and it depends on the size of the patient. If the number of voxels in PE direction is deficient and there occur tissue outside of FOV, it leads to aliasing artifact in the MR image. The final imaging time was 13 ± 2.5 min both for transverse and for coronal imaging, depending on the size of the volunteer. Utilized parallel imaging acceleration factor was 2.

Number of bins was optimized to improve the image quality of the final 4D‐MR image. The self‐developed algorithm was run with bin values of 4–20 and the number of missing slices were calculated.

### 2.4 Phantom test

The 4D‐MRI method was tested and validated with a self‐developed deformable 4D phantom. A deformable phantom for 4D imaging tests was constructed by combining self‐made deformable tissue‐equivalent material and a commercial programmable motor unit from the 4D phantom (CIRS, Model 008A). A phantom was prepared by a 3D‐printed rigid, hollow, and rectangular shell that was filled with silicone gel, an air‐filled balloon and plastic pellets forming a contrast in the MR image. The shell was left open on one side leaving one surface of the flexible material free for deformation. The purpose was to mimic liver–lung interphase with the phantom. The pellets were used as small targets (diameter = 6 mm) to be tracked three‐dimensionally during the simulation of respiratory motion. A motor and piston part of the 4D phantom was employed to produce a controlled movement pattern in the phantom. The piston was directed in the “SI” direction at the center of the flexible surface of the phantom. The maximal movement was observed at the flexible surface of the phantom and the movement was damped as going deeper in the phantom.

The 4D phantom was imaged with the 4D protocol in MRI using T2‐w SSFSE sequence interleaved with 1D navigator (1.5 T GE Optima 450w GEM) and in CT (Siemens Somatom Confidence RT). Imaging was made with different input transformation signals; the shape of cos^6^(x) with 10 and 15 mm displacements with frequency of 7.08 cycles/min. 4D‐CT images were used as a reference image to validate the 4D‐MRI protocol. The MRI and CT images were sorted into 10 bins to get comparable 4D images. Resulted 4D images were compared and center location of the phantom surface was measured with tools in 3D Slicer software.^22^

## 3 Results

### 3.1 Sequence optimization

The imaging parameter TR and the combination of slice thickness, images/slices, and imaging time were optimized. TR was tested with range from 400 to 1500 ms. With lower TR (TR = 400 ms), there occurred remarkable noise in the navigator data. The noise caused inaccuracy to the surrogate respiratory signal. With higher TR, a sampling problem was observed as the frequency of data acquiring was decreased. When TR was increased, the imaging time was increasing excessively. TR 500–550 ms was found feasible for our purpose.

The echo time TE was chosen in order to get sufficient T2‐w MR image contrast, reasonable value being were TE = 80 ms. Slice thickness was balanced between resolution and imaging time, and the slice thickness of 3 mm enables observing small tumors. Number of images/slices was optimized to be as low as possible to optimize the imaging time without increasing the amount of missing data. With low number of slices per location the amount of missing data was unacceptably high, therefore, the value of parameter was increased to 20, which proved to be optimal in relation with missing data and imaging time. Increasing the number of slices per location increases linearly the imaging time. The number of images/slices = 20 per location for reasonable imaging time and quality of data points.

The 5 mm image quality did not meet the requirements of RTP, and, therefore it was excluded. Acquisition with 2 mm slice thickness had good image quality but the imaging time was increased excessively. Images with 3 mm thickness had suitable image quality for RTP, with reasonable imaging time and therefore it was chosen as a feasible compromise to the protocol. Image resolution was 1.33–1.64 × 1.33–1.64 mm^2^ × 3 mm in coronal acquisition plane, 1.17–1.41 × 1.17–1.41 mm^2^ × 3 mm in transversal plane and 1.33 × 1.33 mm^2^ × 3 mm in sagittal plane. When slice thickness was increased 1 mm, the imaging time decreased approximately 20%.

**Fig 3:**
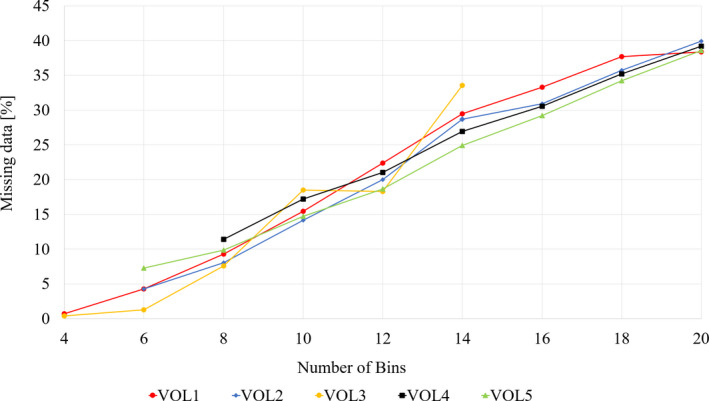
Proportion of missing data as a function of the number of bins for five volunteers (VOL = volunteer). The amount of missing data increases monotonically as the number of bins is increased

**Fig 4:**
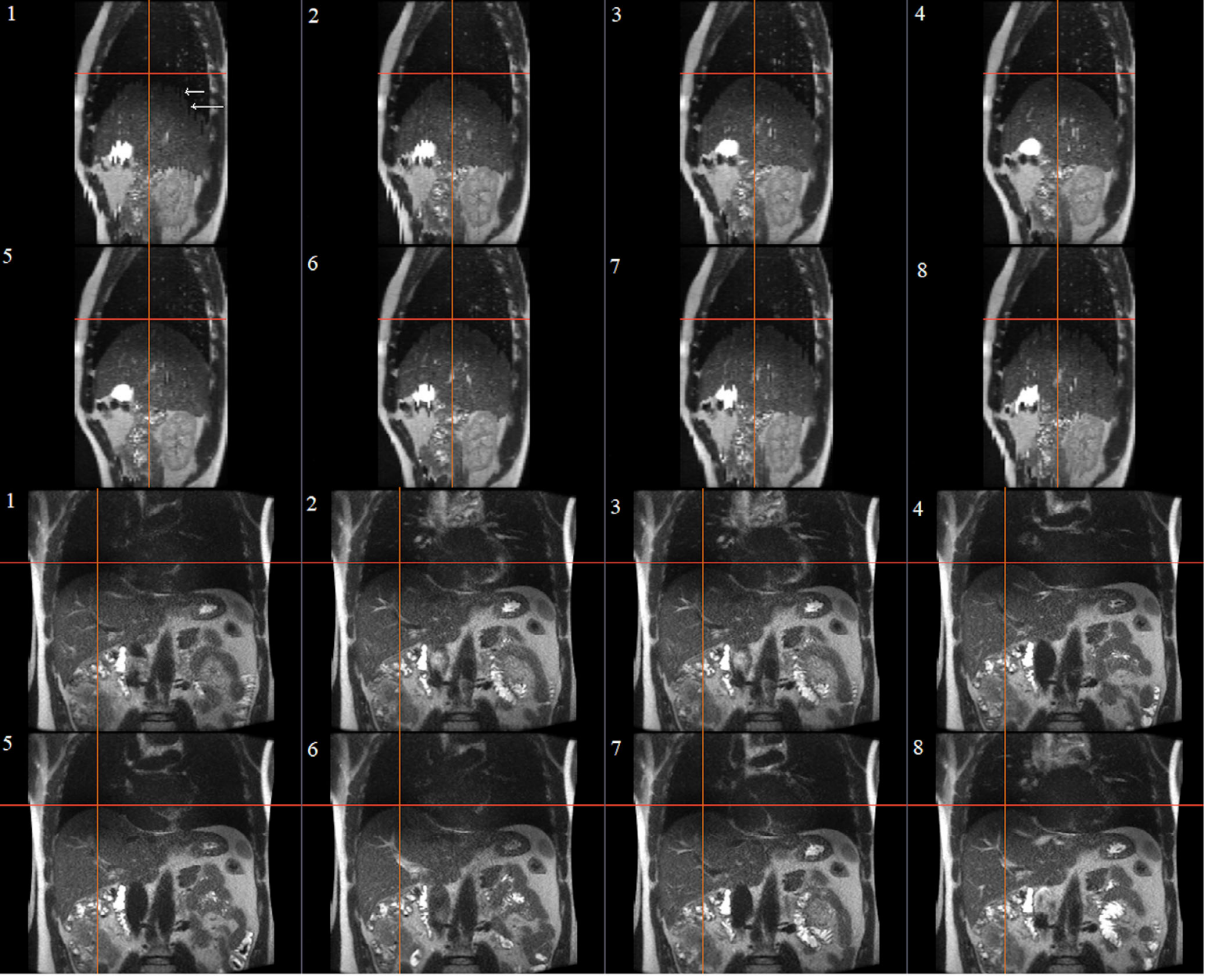
T2‐w SSFSE sagittal (above) and coronal (below) plane MR images from abdomen area (coronal image acquisition) in eight respiratory phases. Images from expiration phases (phase 4,5) has fewer stitching artifacts (marked with arrows at inspiration, phase 1) caused by irregular breathing of volunteer (VOL1). Maximum displacement of the liver dome between the inspiration and expiration phases was 10.6 mm

The 4D sorting algorithm was tested with variable number of bins (4–20), and as a measure of data quality, the number of empty slices were calculated. The results are shown in Fig. 3. With the number of bins ≤10, the data loss is below 18 %. The number of bins = 8 was chosen to reconstruct the 4D‐MR image of healthy volunteers, since it has low amount of stitching artifact (Fig 4, visually observed) and low number of missing data. Interestingly, the different curves have similar shapes, independent from the volunteer imaged.

The imaging sequence results in adequate soft‐tissue contrast in abdomen area and different tissues (fat, muscle, liver, and lung) can easily be visualized (see Fig. 4) with similar quality to the breath‐hold technique. The 4D‐MR images of all five volunteers were visually evaluated, and it was observed that the 4D‐MR images have corresponding image quality as in Fig. 4.

### 3.2 Phantom tests

**Fig 5:**
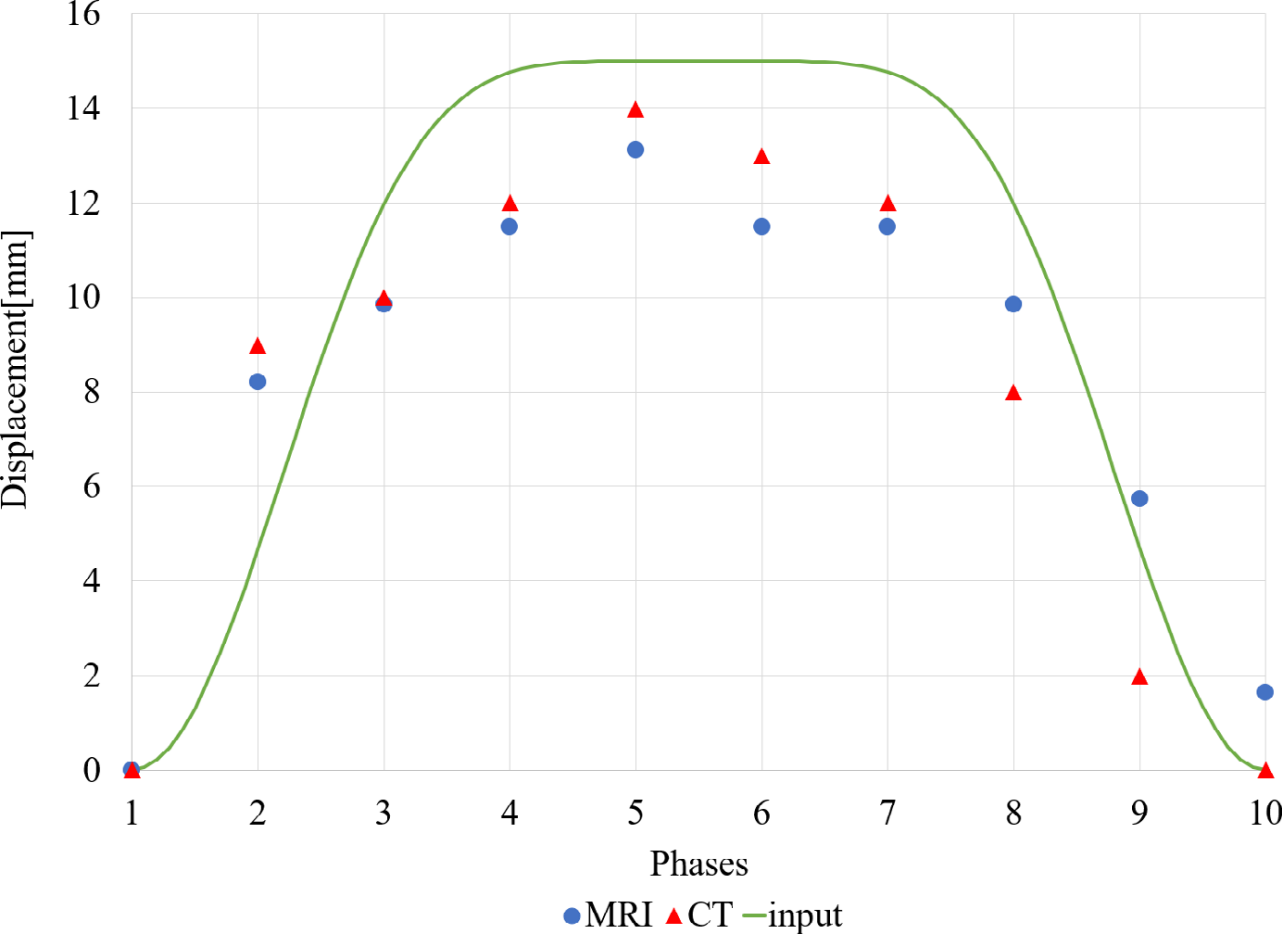
4D phantom displacement as a function of phases. Measured deformations of the 4D‐MRI and 4D‐CT from the surface of the phantom. The input signal was cos^6^(x) with 15 mm displacement and 7.08 cycles/min frequency. The maximum observed displacement as measured from MRI was 13 and 14 mm with CT. The deformable nature of the phantom causes that the resulted surface displacements are shallower in MRI and CT than the input displacement

Phantom result showed that output deformations measured from the surface of the phantom are compatible with the input movement. Displacements of the surface of the phantom were measured from the 4D‐MRI and 4D‐CT images (Fig. 5). Displacements measured from the images were compared with the shape of the actual “input” movement of the 4D phantom piston, and it was observed that both image modalities visualize 4D motion with a similar accuracy. In case of the maximum displacement of the input movement was 15 mm, the resulted maximum deformations deviated less than 2 mm in 4D‐MRI and 1 mm in 4D‐CT from the input. All displacements of the 4D‐MRI and the 4D‐CT were compared to each other. Phantom results showed that the developed method is able to detect the motion with accuracy of 1.2 mm mean, 1.1 mm standard deviation, compared to 4D‐CT when the frequency of the movement was less than 7.08 cycles/min.

## 4 Discussion

The 4D‐MRI method of liver was developed^20^ and optimized. In addition, self‐developed MATLAB algorithm was produced for data processing. The resulted 4D‐MR image models the liver motion three‐dimensionally in one respiratory cycle with an acceptable imaging time and sufficient temporal and spatial accuracy. The method fulfills clinical prerequisites and method was successfully tested and validated with a self‐developed phantom.

Navigator data consist of 1D images collected from the liver–lung interphase. The navigator is centered manually on the dome of the liver by using the 2D localizer images, which are taken during free breathing of volunteer. Navigator image data have a certain amount of noise caused by physiological motion, thus the processing of navigator data requires filtering before the respiratory surrogate signal is reconstructed.^23^ According to Wang et al. (1996),^23^ the least square algorithm removes noise and profile deformations from navigator data. We were able to optimize imaging sequence to reduce noise and therefore to determine the respiratory signal by thresholding the navigator signal.

Our method was developed with healthy volunteers and special breathing instructions were not provided. Due to free breathing, there may occur coughing or other natural undesired irregular events. These short‐term events are recognized and excluded from 4D liver model since the purpose is to model averaged liver motion. The detection of undesired event needs to be considered especially for future studies, when patients are imaged. The five healthy volunteers were not representative of typical RTP patients as they were not *in vivo* pathology cases and because of their age range (27–52 years). Patient might have even more irregularities with their breathing since their physical condition will be variable. In addition, it needs to be considered that the sampling is approximately 2 Hz, and therefore short‐term events such as fast inspiration might get unobserved during MRI data collection. Imaging time should be clinically acceptable and liver drifting during imaging should be avoided. The imaging time in this research is acceptable for healthy volunteers; however, decreasing of the imaging time should be more considered in future studies with patients. The imaging time could be decreased, for example, by leaving out the other imaged acquisition plane and/or by decreasing the imaging FOV or number of slices to consist of smaller area of abdomen. The slice thickness of 5 mm increase image SNR, but would create partial volume artifacts. Increasing the slice thickness would improve the missing slice artifact and intra‐bin variability. The 3 mm slice thickness showed reasonable spatial resolution and even small details as veins and their motion were clearly detectable from the volunteer’s final 4D‐MR image.

The phantom results showed that the standard 4D‐CT and the developed 4D‐MR images have a comparable performance. The 4D image generation processes are different between CT and MR images, which may be the reason why there occur few varying points in Fig. 5. The mean accuracy was found to be 1.1 mm and the MR and CT images obey the shape of the cos^6^(x) signal. In Fig. 5, the time resolution is not correct because of an unmeasurable delay between input and respond. The phantom response has lower amplitude than the input that might be caused by the elastic nature of the phantom. It is also possible that our measurements had minor setup errors. Imaging parameters such as slice thickness and acquisition direction are different between CT and MRI. The CT was imaged helically with low pitch value and the MRI was imaged in interleaved acquisition order. All differences in imaging method and 4D image processing may lead to variations in final 4D images. The phantom was imaged in the MR and CT scanners with the same setup; however, the phantom was moved between imaging modalities. The self‐developed elastic phantom may act differently between measurements; the phantom material may recover differently back to steady state after the input. More comprehensive phantom test should be continued in future studies to statistically validate 4D motion of inner small objects of the phantom.

There are several different types of 4D‐MRI methods published but commercial applications are not yet available.^2^ There are no publications with the same approach as our method. Therefore, direct comparison between studies is not fully possible. Our method utilizes phase binning method to get probabilistic information about the spatial state of the liver. The information enables method utilizing in delineation in RTP. Number of bins requires compromises between two undesired characteristics: number of missing slices and intra‐bin variability. Our research showed that when MR images of liver are sorted in eight respiratory phases, clinically accurate 4D images are produced with low intra‐bin variability and low proportion of missing data (<18%).

The developed 4D method suffers from missing data problem caused by data sampling. In our method, missed slices were replaced with slice from previous bin, which may reduce the quality of the resulted image. The replace method was used to mimic the real situation without additional interpolation that may cause image blurring artifact. Other studies have used image averaging to interpolate missing slices.^19^ Van de Lindt et al. (2018)^24^ missing data were interpolated using iterative interpolation algorithm, which uses both time and space to predict the missing values based on discrete cosine transforms. Missing data problem does not occur with cine imaging that is used in some papers.^9^ Cine imaging reduces the risk of missing slices since the same location is imaged until whole respiratory cycle is acquired and only then moving to next location. However, the imaging time is longer in cine‐mode than in interleaved or in sequential acquisition.^9^, ^10^, ^11^ The interleaved acquisition order ensures longer relaxation time before next excitation, which reduces cross talk and improves image contrast.^2^

The quality of the resulted 4D image varies for each imaged volunteer. The quality of the 4D‐MR image is mainly reduced by the stitching artifact caused by the irregularity of volunteer’s breathing. Our results show that stitching artifact is more visible in inspiration phase (Fig 4). Van de Lindt et al. (2018)^24^ observed same in their research. Irregular breathing is the main disturbing event in all respiratory‐induced imaging methods.^2^ Stitching artifact may be decreased with additional amplitude range selection from respiratory signal. Additional amplitude approach would increase the number of missing slices in our method since more data would be excluded. The stitching artifact could be also minimized with averaging mid‐ventilation^9^ or mid‐position^19^ approach.

Freedman et al. (2018)^19^ used the 2D T2‐w HASTE MRI sequence in axial and sagittal acquisition planes, 10–30 slices/locations, 5 mm slice thickness with a derived respiratory surrogate to create retrospectively mid‐position “super‐resolution” (1 × 1 × 1 mm^3^) 4D‐MR image sorted in eight bins. Their research had similar imaging parameters as in our study. Their results reduced the stitching artifact and the missing data artifact when deformable image registration and super‐resolution reconstruction was used. This two‐image plane method resulted more accurate 4D‐MR images than our one plane method, since the final image had more exact resolution and less artifacts. The deformable image registration method will be utilized in our future studies to build deformable liver model and to get 4D‐MR image with detailed resolution. We are currently studying the visible lesions of real patients and planning to publish the results soon.

The 4D image can model the motion of the liver and therefore, it enables tracking motion of the liver. The image models the liver motion accurately especially in expiration phase where the stitching artifact caused by irregular breathing is minimal. Our method models the liver three‐dimensionally in eight respiratory phases with more accurate resolution and larger FOV than most of the published studies.^2^ The resulted images had also illustrative contrast, thus small details such as veins can be observed.

## 5 Conclusions

A novel method of 4D‐MRI of liver was developed in order to produce dynamic MRI sequence for planning of SBRT treatments of liver metastasis. The developed method has promising features to meet clinical requirements, and achieve acceptable resolution and lowest degree of artifacts to resulted images. The 4D‐MRI method robustly demonstrates the 3D motion of the liver in one respiratory cycle. The method was successfully tested with five volunteers, and with a 4D phantom test it reached similar accuracy than the reference 4D‐CT method.

## Author’s contribution

Henna Kavaluus is the corresponding author, and involved in data collection, data analysis, and manuscript writing. Tiina Seppälä involved in data collection, data analysis, and reviewing the manuscript. Lauri Koivula involved in data collection and reviewing the manuscript. Eero Salli involved in designing the study, data analysis, and reviewing the manuscript. Juhani Collan involved in reviewing the manuscript. Kauko Saarilahti and Mikko Tenhunen involved in designing the study and reviewing the manuscript.
